# Nighttime blood pressure decline as a predictor of renal injury in patients with hypertension: a population-based cohort study

**DOI:** 10.18632/aging.101873

**Published:** 2019-07-05

**Authors:** Dong Cheng, Yi Tang, Haiyu Li, Yunpeng Li, Haiqiang Sang

**Affiliations:** 1 Department of Cardiology, the First Affiliated Hospital of Zhengzhou University, Zhengzhou 450000, Henan Province, China

**Keywords:** hypertension, biomarkers, glomerular filtration rate, nighttime blood pressure decline

## Abstract

We explored whether the nighttime blood pressure (BP) decline predicts renal function decline in a population-based cohort with primary hypertension. We measured the baseline ambulatory BP and glomerular filtration rate (GFR) in a cohort of 1,042 primary hypertensive patients. We repeated the GFR measurements and calculated the rate of GFR decline after a median follow-up of 5.8 years. The estimated GFR (eGFR) declined by −0.23 to −0.20 mL/min per year as the nighttime systolic BP (SBP), diastolic BP (DBP), and mean BP decline rates increased by 1% (*P* < 0.001). In the fully adjusted model, the nighttime SBP, DBP, and mean BP were all related to a steeper rate of eGFR decline by −0.25 to −0.22 mL/min per 1% increase. The adjusted multivariable results indicated that the odds of an eGFR decline were reduced by 46% when the nighttime SBP decline rate increased by 1% (OR= 0.54, 95% CI: 0.46–0.62). The restricted cubic spline model indicated a non-linear dose-response relationship with the nighttime SBP, DBP, and mean BP. Nighttime BP may be an important biomarker of renal function injury in hypertensive patients.

## Introduction

Hypertension is one of the most common chronic diseases and the most important risk factor for cardiovascular disease [1]. The major complications of hypertension, such as coronary heart disease, heart failure, stroke, and chronic kidney disease (CKD), cause a heavy burden for families and society [2]. With social and economic development, the incidence of hypertension is increasing, and it has become an important public health problem of the 21^st^ century worldwide [3]. The prevalence of CKD is also increasing dramatically worldwide. CKD progresses to end-stage renal disease requiring renal replacement therapy [4,5], which markedly alters the quality of life of CKD patients. Primary hypertension is the most common cardiovascular disease and one of the most important risk factors for CKD [6]. Therefore, a better understanding of the modifiable risk factors of early renal dysfunction is necessary. For example, awareness of glomerular hyperfiltration and hypofiltration, leading to early detection and prevention, might alleviate the future burden of CKD and associated complications in patients with hypertension.

Blood pressure (BP) follows a circadian rhythm with 10–15% lower values during the night than during the day [7]. Daytime BP is more closely related to target organ damage than nighttime BP [8,9]. However, some studies also found that a blunted decrease in the nighttime BP (absence of a nocturnal BP dip) was associated with target organ damage in hypertensive patients, including left ventricular hypertrophy and other cardiovascular events [10,11]. Moreover, nighttime BP and nocturnal BP fall are more strongly correlated with left ventricular hypertrophy than daytime BP or 24-hour average BP [12,13]. A study with a large sample size suggested that the absence of a nighttime BP dip increased the risk of all-cause mortality and cardiovascular events, and the increased risk was higher than that due to the 24-hour BP. Furthermore, some results indicated that non-dipping nighttime BP caused deterioration in CKD [14]. So far, this phenomenon has been elucidated only in patients with mild proteinuria. Therefore, this study explored whether the nighttime BP decline predicts renal function decline in a population-based cohort with primary hypertension.

## RESULTS

### Baseline characteristics

**Figure 1:**
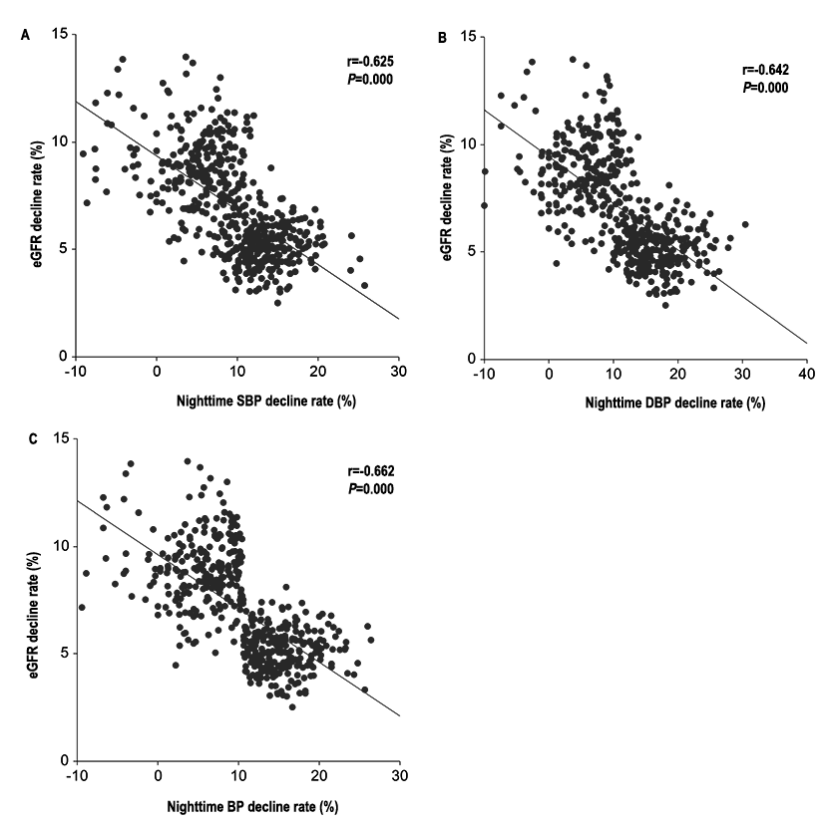
Scatter diagram of relationship between nighttime SBP (**A**), nighttime DBP (**B**) and nighttime BP (**C**) decline rate.

Table 1 presents the baseline characteristics of the entire cohort according to the mean nighttime BP decline rate. The patients with a nighttime BP decline rate≤mean were more often female, had history of smoking, were older, and had higher total cholesterol, sCr, and ALT levels (*P* < 0.05). Significant differences were also observed in RBC, hemoglobin, aldosterone, and angiotensin II. There was no significant difference in the baseline eGFR level (*P* = 0.931) between the two groups. The absolute eGFR change and rate were significantly lower in the patients with a lower BP decline rate than in patients with a greater BP decline rate (*P* < 0.05). Table 2 summarizes the office BP and 24-h ambulatory BP. The group with a low nighttime BP decline rate had a higher office SBP, day mean DBP, and mean nighttime SBP and DBP. There was a negative relationship between the eGFR decline rate and nighttime SBP decline rate (r = −0.625, *P* < 0.001, Figure 1A), nighttime DBP decline rate (r = −0.642, *P* < 0.001, Figure 1B) and nighttime BP decline rate (r = −0.662, *P* < 0.001, Figure 1C).

**Table 1:** Baseline characteristics of the population according to nighttime blood pressure decline rate.

Baseline characteristic	Nighttime blood pressure decline rate≤Mean	Nighttime blood pressure decline rate>mean	t/χ^2^/μ	*P*
N	491	551		
Sex (male, n (%))	283(57.6%)	361(65.5)	6.829	0.009
Age, y	47.4±7.9	46.4±7.7	2.096	0.036
Body mass index, kg/m^2^	25.7±3.3	26.0±3.5	-1.471	0.142
Waist circumference, cm	95.1±9.5	93.8±10.1	2.133	0.033
Smoking, n (%)	112(22.8%)	40(7.3%)	50.395	0.000
Drinking, n (%)	139(28.3%)	141(25.6%)	0.977	0.323
Physical exercise, n (%)			5.620	0.060
Never	22(4.5%)	35(6.4%)		
Low intensity	189(38.5%)	240(43.6%)		
High intensity	280(57.0%)	276(50.0%)		
Triglyceride, mmol/dL	1.9±0.5	1.8±0.5	1.944	0.052
HDL-cholesterol, mmol/dL	1.1±0.2	1.1±0.2	-1.855	0.064
LDL-cholesterol, mmol/dL	3.2±0.8	3.1±0.9	1.162	0.246
Total cholesterol, mmol/dL	5.4±0.9	5.2±0.9	3.581	<0.001
Fasting glucose, mmol/dL	5.7±0.7	5.6±0.6	1.601	0.110
Postprandial glucose, mmol/dL	7.6±2.4	7.4±2.1	1.087	0.278
Serum creatine, mmol/dL	70.7±13.5	69.0±12.6	-2.10	0.036
Uric acid, mmol/L	342.6±49.6	343.4±46.9	-0.252	0.801
Urinary albumin-creatinine ratio, mg/mmol	49.2±44.4	44.6±45.1	1.656	0.098
Blood urea nitrogen, mmol/L	4.8±1.1	4.8±1.2	-0.304	0.761
ALT*, U/L	29.0±23.9	32.4±25.0	-2.21	0.027
AST*, U/L	25.5±11.9	25.1±9.9	0.464	0.643
White blood cell, ×10^9^	6.5±1.8	6.3±1.7	0.885	0.376
Red blood cell, ×10^12^	4.9±0.4	5.0±0.5	-3.365	0.001
Hemoglobin, g/L	147.6±16.4	152.1±14.9	-3.645	<0.001
Red cell distribution width, %	12.9±0.9	12.8±0.8	1.899	0.057
Platelet count, ×10^9^	239.1±55.1	236.0±51.2	0.941	0.347
High-sensitive CRP, mg/L	2.7±5.9	2.3±3.4	1.358	0.175
Plasma renin activity, ug/lh	3.3±3.8	3.4±3.8	-0.044	0.965
Aldosterone, mol/L	0.2±0.1	0.2±0.0	2.546	0.011
Angiotensin II	77.9±31.7	72.8±26.2	2.814	0.005
Medication, n (%)				
β-blocker	70(14.3%)	74(13.4%)	0.149	0.699
ACE inhibitor	101(20.6%)	116(21.1%)	0.037	0.848
A2 blocker	111(22.6%)	98(17.8%)	3.764	0.052
Calcium blocker	327(66.6%)	385(70.0%)	1.286	0.257
Diuretic	23(4.6%)	21(3.8%)	0.489	0.484
Lipid-lowering medication	68(13.8%)	55(9.9%)	3.730	0.053
GFR, ml/min per 1.73m^2^	84.1±17.2	84.2±17.5	-0.087	0.931
GFR<60 ml/min per 1.73m^2^	24(2.5%)	11(2.7%)	3.380	0.094
Absolute GFR change ml/min per year	-3.6±1.8	-1.4±2.0	-3.377	<0.001
Absolute GFR rate, %	-8.9±1.7	-5.1±1.0	-44.582	<0.001
GFR after follow-up, ml/min per 1.73m^2^	76.8±17.2	80.0±17.4	-2.931	0.003

*ALT: glutamic-pyruvic transaminase, AST: glutamic oxalacetic transaminase, GFR: Glomerular filtration rate; Mean of nighttime blood pressure decline rate 9.42%.

**Table 2:** Office blood pressure and 24h ambulatory blood pressure according to nighttime blood pressure decline rate.

Baseline blood pressure	Nighttime blood pressure decline rate≤mean	Nighttime blood pressure decline rate>mean	t	*P*
Office blood pressure				
Systolic, mmHg	153.9±12.7	151.1±10.9	3.886	<0.001
Diastolic, mmHg	99.8±9.1	99.9±8.6	-0.276	0.783
Mean blood pressure, mmHg	117.8±8.9	117.0±8.1	1.612	0.107
Heart rate, n/min, mmHg	78.1±9.5	78.9±9.4	-1.277	0.204
24h ambulatory blood pressure, mmHg				
Day mean systolic, mmHg	140.0±11.7	140.1±10.4	-0.119	0.905
Day mean diastolic, mmHg	90.3±10.1	92.1±7.9	-3.205	0.001
Day heart rate, n/min	76.9±8.6	79.9±8.8	-5.584	<0.001
Nighttime mean systolic, mmHg	133.1±12.5	121±9.6	17.302	<0.001
Nighttime mean diastolic, mmHg	85.1±10.2	76.8±7.5	15.094	<0.001
Nighttime heart rate, n/min	65.1±8.0	65.1±7.9	-0.057	0.954
Nighttime systolic pressure decline, %	106.7±9.6	108.1±7.8	-2.251	0.025
Nighttime diastolic pressure decline %	101.1±7.3	91.6±7.3	17.553	<0.001
Nighttime blood pressure decline rate, %	5.4±4.1	15.2±3.3	-42.910	<0.001

### Nighttime BP decline and eGFR change rates

The eGFR declined more rapidly in the low nighttime BP decline rate group than in the high nighttime BP decline rate group. We examined the nighttime SBP, DBP, and mean BP declines rate in separate mixed linear models with stepwise addition of factors. For Model 1, the eGFR declined by −0.23 to −0.20 mL/min per year when the nighttime SBP, DBP, and mean BP decline rates increased by 1% (*P* < 0.001, Table 3). The same results were observed in Model 2. In the fully adjusted model with all of the variables, the nighttime SBP, DBP, and mean BP were all related to a rate of eGFR decline steeper by −0.25 to −0.22 mL/min per 1% increase.

**Table 3:** Association between baseline nighttime blood pressure decline rate and GFR change rates in linear mixed regression analyses.

Independent variable	Model 1			Model 2			Model 3		
	*β*	95%CI	*P*	β	95%CI	*P*	*β*	95%CI	*P*
Nighttime SBP decline rate, per 1% increase	-0.23	-0.25: -0.22	<0.001	-0.22	-0.25: -0.20	<0.001	-0.22	-0.26: -0.19	<0.001
Nighttime DBP decline rate, per 1% increase	-0.20	-0.24: -0.19	<0.001	-0.20	-0.22: -0.18	<0.001	-0.20	-0.22: -0.17	<0.001
Nighttime BP decline rate, per 1% increase	-0.23	-0.25: -0.22	<0.001	-0.25	-0.27: -0.22	<0.001	-0.25	-0.27:-0.22	<0.001

Model 1: adjusted for baseline GFR, sex, age, BMI, waist circumstance, smoking, drinking, physical exercise and medication (β-blocker, ACE inhibitor, A2 blocker, Calcium blocker, Diuretic Lipid-lowering)

Model 2: adjusted Model 1 and in addition triglyceride-cholesterol, LDL-cholesterol, total cholesterol, fasting glucose, postprandial glucose, serum creatine, uric acid, urinary albumin-creatinine ratio, blood urea nitrogen, ALT, AST, white blood cell, red blood cell, Hemoglobin, red cell distribution width, platelet count, office mean blood pressure, 24 h mean blood pressure

Model 3: adjusted Model 2 and in addition, high-sensitive CRP, plasma renin activity, aldosterone, angiotensin II

### Risk of a rapid eGFR decline

We explored the risk of a rapid eGFR decline defined as a rate of change less than the mean value in the multiple logistic regression models adjusting for the parameters as in the linear mixed models. The unadjusted univariate analyses are presented in Supplementary Material. Table 4 shows the adjusted multivariable results. The univariate analyses indicated that the nighttime SBP decline rate was negatively associated with the risk of eGFR decline (OR = 0.71, 0.99, *P* < 0.001, Table S1). As presented in Table 4, increased nighttime SBP, DBP, and mean BP decline rates were associated with lower risks of eGFR decline in all models. In the fully adjusted model, the odds of an eGFR decline were reduced by 46% when the nighttime SBP decline rate increased by 1%.

**Table 4:** Baseline Nighttime pressure decline rate and GFR change rates in logistic regression.

Independent variable	Model 1			Model 2			Model 3		
	Odds ratio	95%CI	*P*	Odds ratio	95%CI	*P*	Odds ratio	95%CI	*P*
Nighttime SBP decline rate, per 1% increase	0.58	0.55-0.62	<0.001	0.53	0.45-0.62	<0.001	0.54	0.46-0.62	<0.001
Nighttime DBP decline rate, per 1% increase	0.62	0.59-0.66	<0.001	0.45	0.36-0.57	<0.001	0.46	0.37-0.58	<0.001
Nighttime BP decline rate, per 1% increase	0.53	0.48-0.57	<0.001	0.31	0.22-0.45	<0.001	0.32	0.22-0.45	<0.001

Model 1: adjusted for baseline GFR, sex, age, BMI, waist circumstance, smoking, drinking, physical exercise and medication (β-blocker, ACE inhibitor, A2 blocker, Calcium blocker, Diuretic, Lipid-lowering medication)

Model 2: adjusted Model 1 and in addition triglyceride-cholesterol, LDL-cholesterol, total cholesterol, fasting glucose, postprandial glucose, serum creatine, uric acid, urinary albumin-creatinine ratio, blood urea nitrogen, ALT, AST, white blood cell, red blood cell, Hemoglobin, red cell distribution width, platelet count, office mean blood pressure, 24 h mean blood pressure

Model 3: adjusted Model 2 and in addition, high-sensitive CRP, plasma renin activity, aldosterone, angiotensin II

**Figure 2:**
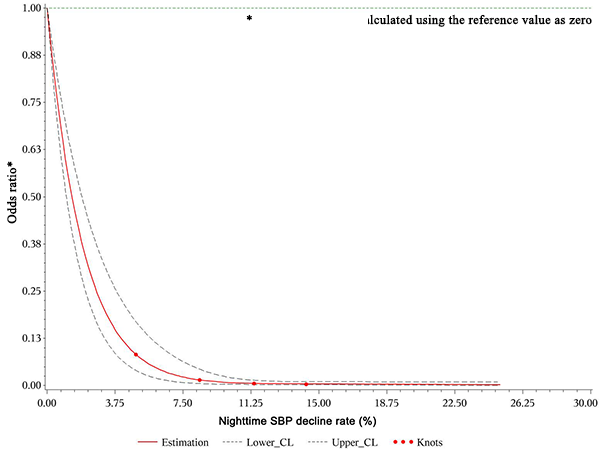
**Restricted cubic spline plot of risk of rapid eGFR decline from nighttime SBP decline rate>0.** A positive rate of change means that the night BP declined and this decline reduced the odds ratio of renal injury during follow-up.

**Figure 3:**
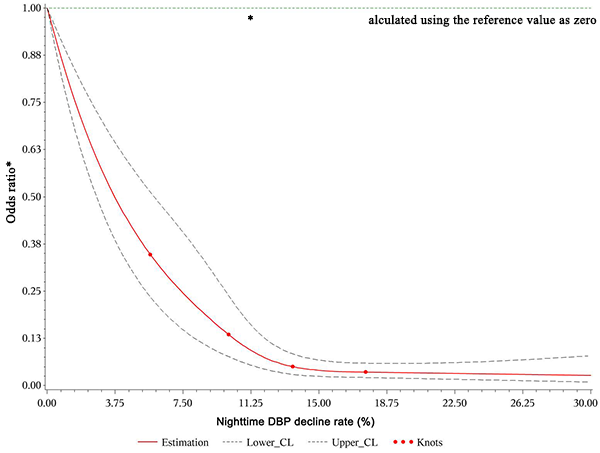
**Restricted cubic spline plot of risk of rapid eGFR decline from nighttime DBP decline rate>0.** A positive rate of change means that the night BP declined and this decline reduced the odds ratio of renal injury during follow-up.

**Figure 4:**
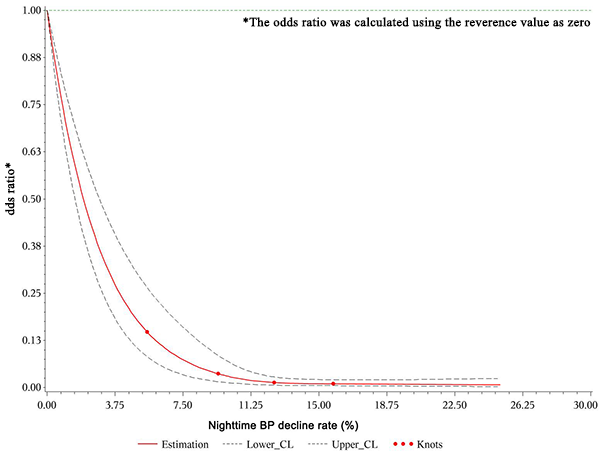
**Restricted cubic spline plot of risk of rapid eGFR decline from nighttime mean BP decline rate>0.** A positive rate of change means that the night BP declined and this decline reduced the odds ratio of renal injury during follow-up.

We also built dose-response relationships between the nighttime SBP, DBP, and mean BP decline rates and the eGFR decline rate based on the restricted cubic spline method (four knots: 5.68, 10. 13.54, and 17.58; reference value 0). The restricted cubic spline methods are plotting the odds ratio. First, what we need to know is that: the BP decline rate <0 when day BP< nighttime BP, the BP decline rate=0 when day BP=nighttime BP, and the BP decline rate>0 when day BP>nighttime BP according the formula. In the Figure 2-Figure 4, we treated the BP decline rate=0 as the reference level and we plotted the odds ratio in patients with BP decline rate>0 and BP decline rate<0, respectively. Taking the Figure 2 as example, the odds ratio<1 because the nighttime SBP decline rate>0(day SBP>nighttime SBP), which means the nighttime SBP is lower than day SBP. The decreased the nighttime SBP reduced the risk of GFR decline (OR>1). After adjusting for all variables, the restricted cubic spline model indicated non-linear dose–response relationships between the nighttime SBP (Figure 2), DBP (Figure 3), and mean BP (Figure 4) decline rates and the eGFR decline per year where the reference value is 0.

## DISCUSSION

This study found that nighttime BP decline, a non-invasive, accessible cardiovascular biomarker, was an independent predictor of renal function decline in patients with hypertension. A decline of 0.25 mL/min per year per 1% increase in nighttime BP was clinically important. The logistic regression showed similar results. The results were still robust after excluding the history of medicine usage. Therefore, renal function injury in hypertensive patients may explain the results.

Twenty-four-hour ambulatory BP monitoring (ABPM) is widely used in clinical practice [15]. The ambulatory 24-hour BP can predict the prognosis better than the office BP and the average nighttime sleep BP is generally a better predictor of adverse cardiovascular outcomes than the average daytime BP on ABPM [13,16]. There is normal circadian BP variability, with higher levels during daytime and a 10–20% BP fall at night. O’Brien first reported that hypertensive patients with a blunted nocturnal BP fall had a greater prevalence of strokes and called these patients non-dippers, in contrast to normal dippers [17]. Subsequently, several prospective studies reported that the nighttime BP fall was associated with the prognosis of hypertensive patients [18,19]. However, these reports were not consistent, possibly because of differences in methodology, study populations, sample sizes, and end points. More importantly, the previous studies did not adjust the analyses for the average 24-hour BP or did not examine the nocturnal BP decline as a continuous variable [20]. To our knowledge, the nighttime BP decline rate has not previously been explored as a potential risk predictor for renal function decline in hypertension. Li *et al*. monitored the ambulatory BP and followed health outcomes in 588 Chinese CKD patients and found that a higher nighttime BP load, especially the nighttime DBP load, was associated with a poorer prognosis in Chinese non-dialysis CKD patients [21]. Ruiz-Hurtado *et al*. examined the quantitative differences in nighttime SBP across albuminuria levels in 16,546 patients with and without diabetes and CKD and reported that albuminuria in hypertensive patients is accompanied by a strikingly higher nighttime SBP, particularly in those with diabetes with very high albuminuria and a low eGFR [22]. Our results extend these findings by showing that the nighttime BP decline rate is also related to an accelerated GFR decline in hypertensive patients.

The pathophysiological mechanism of the association between reduced nocturnal BP and worsening cardiovascular outcomes has not been confirmed, although it has been widely discussed [23]. Hypotheses include a night autonomic nerve disorder, which is beneficial to sympathetic hyperactivity, baroreceptor sensitivity change [24], increased myocardial repolarization [25], increased salt sensitivity or renal dysfunction, nocturnal overload capacity [26], need for a higher BP to maintain a natriuretic night, sleep apnea or poor sleep quality, high aldosteronism state, increased arterial stiffness [27], chronic low-grade inflammation, endothelial dysfunction, and orthostatic hypotension in the day [28]. The pathophysiological mechanism of the poor prognostic effect of extreme tilt mode according to the antihypertensive state is completely unknown, but we speculate that it might include orthostatic hypertension, an exaggerated BP surge in the morning, BP variability increases, or increased hardening of the arteries [29,30]. These might be partly attenuated by antihypertensive treatment.

A change in nighttime BP means an abnormal circadian rhythm of BP. The abnormal circadian rhythm itself does not cause symptoms or signs and it is difficult to detect with routine measures. For hypertensive patients, an analysis of their BP circadian rhythm can better identify information related to the occurrence of organ damage and clarify the relationship between BP biological rhythm and organ damage. Therefore, the ABPM becomes an important tool for monitoring the occurrence and development of early renal function injury. The ABPM can be used to detect patients with high-filtration renal damage and hypertension to guide drug treatment to treat and prevent the progress of CKD. The application of 24-hour dynamic BP monitoring should improve the diagnosis of early renal damage, and nighttime BP monitoring may become an important indicator of the occurrence and development of hypertensive renal damage. In addition, restoring the circadian rhythm of BP is as important as controlling the average BP, which is the goal of standard hypertension treatment and reduces the incidence of cardiovascular and cerebrovascular complications.

The main strength of our study was that we evaluated the 24-hour ambulatory BP, which might be a better measure of BP than the office BP used in other studies. The ABPM enables more accurate BP measurement. We were also able to adjust the analyses for important determinants of BP, such as physical activity, drug use, and hemoglobin concentration, to reduce potential confounding. Finally, we excluded patients with severe cardiovascular diseases, such as heart failure, to reduce their possible influence. The main limitation of the study was that the GFR was estimated using a formula instead of using the iohexol clearance, which excludes confounding from non-GFR-related factors. The inclusion of only a Chinese population limits the generalizability of our results to other ethnic and age groups. For the nighttime BP decline rate, a repeated renal function examination might have better validated our results. Causality cannot be obtained from an observational study and further research to determine the mechanism is required.

In conclusion, the nighttime BP decline rate was an independent predictor of renal function injury in patients with hypertension. The nighttime BP should be considered when building risk prediction models for renal function decline in hypertension. Future studies should explore the specific mechanism of this relationship.

## MATERIALS AND METHODS

### Study population

This was a single-center, retrospective cohort study of data from the Department of Cardiology, the First Affiliated of Hospital of Zhengzhou University. This study analyzed primary hypertensive patients who were admitted to the hypertension ward or seen as outpatients from November 2007 to October 2009. Hypertension was defined as a systolic BP (SBP) ≥ 140 mmHg, diastolic BP (DBP) ≥ 90 mmHg, or previous use of an anti-hypertensive drug. The baseline glomerular filtration rate (GFR) was measured by the estimation method between 2007 and 2009 and follow-up GFR was obtained between 2013 and 2015. Patients with myocardial infarction, stroke, heart failure cancer, systemic inflammatory disease, hematological disorder, and serious neurological disease were excluded. The study enrolled 1,042 patients between the ages of 30 and 75 years old with baseline and follow-up GFR measurements after excluding 23 subjects who had died. The median observation time was 5.8 years. This study was approved by the Ethics Committee of the First Affiliated Hospital of Zhengzhou University. The research was conducted in accordance with the World Medical Association Declaration of Helsinki, and all subjects provided informed consent.

### Data collection

#### General information

The data collected included demographic and laboratory results at baseline and follow-up. A health questionnaire asked about age, gender, weight, height, waist circumference, comorbidity, smoking, drinking, and physical exercise (three categories: never, low intensity, high intensity) [31]. Antihypertensive medications were obtained from the medical records. Smoking was defined as current smoking or smoked daily previously. Drinking was defined as >2 times a month [32,33].

#### 24-Hour ambulatory BP measurements

At inclusion, the SBP and DBP of each patient were measured automatically every 20 minutes between 6:00 and 21:59 and every 30 minutes from 22:00 to 5:59 for 24 consecutive hours with a calibrated Space Labs 90207 ambulatory BP monitor (ABPM; Space Labs, Issaquah, WA, USA). The 24-hour average SBP and DBP were determined (all time points averaged). A BP series was considered invalid for analysis if ≥ 30% of the measurements were missing, if data were lacking for an interval > 2 hours, if data were obtained while the patients had an irregular rest–activity schedule during the 2 days of monitoring, or if the nighttime sleep period was < 6 or > 12 hours during the ABPM measurements. The nighttime BP decline rate was defined as [(day mean/systolic/diastolic BP) − (night mean/systolic/diastolic BP)] / (day mean/systolic/ diastolic BP). A negative rate of change means that the night BP increased. The mean arterial pressure was estimated as the diastolic BP plus one-third of the pulse pressure.

#### Biochemical examination

We calculated the GFR using the modified MDRD equation [34]. The rate of GFR change was calculated as the (follow-up eGFR-baseline eGFR)/time (between two measurements). A negative change in the estimated GFR (eGFR) indicated a decline in renal function. Hematological parameters were measured on an automated hematology analyzer. Serum creatinine (sCr) was measured using the Jaffe rate-blanked compensated creatinine assay. The fasting blood glucose, sCr, uric acid, urinary albumin–creatinine ratio, blood urea nitrogen (BUN), routine hematology examination (red blood cells [RBC], white blood cells [WBC], hemoglobin, red cell distribution width, and platelet count), high-sensitivity C-reactive protein (hs-CRP), alanine aminotransferase (ALT), aspartate transaminase (AST), aldosterone, angiotensin II, plasma renin activity, and fasting serum lipid status, including total cholesterol, low-density lipoprotein (LDL), high-density lipoprotein (HDL), and triglyceride levels, were also recorded. Body mass index (BMI) was calculated as weight (kg) divided by height squared (m^2^).

### Statistical analysis

The study population was divided into two groups according to the nighttime BP decline rate using a cutoff of 9.42%. For continuous variables, mean ± standard deviation or median (minimum and maximum) was used for statistical descriptions. Differences between groups were tested using the independent-samples *t-*test or Wilcoxon rank-sum test. Categorical variables were expressed as counts and percent, and the χ^2^ test was used to examine differences. Pearson’s correlation coefficient with a scatterplot was calculated to assess the relationship between the eGFR decline rate and nighttime BP decline rate. Missing baseline data was input using multiple imputation. We used two approaches to explore the influence of the nighttime BP decline rate on the eGFR decline. First, a mixed linear model was used with eGFR (continuous variable) as the dependent variable. Second, eGFR was categorized as a dichotomous variable using the mean eGFR decline rate as a cut-off and logistic regression was used. Both the mixed linear and logistic regression models adjusted for several factors. Three models were used: Model 1 adjusted for baseline GFR, sex, age, BMI, waist circumstance, smoking, drinking, physical exercise, and medication usage (β-blockers, ACE inhibitors, A2 blockers, calcium blockers, diuretics, and lipid-lowering medication); Model 2 adjusted for the parameters in Model 1 and triglyceride-cholesterol, LDL-cholesterol, total cholesterol, fasting glucose, postprandial glucose, sCr, uric acid, urinary albumin-creatinine ratio, BUN, ALT, AST, WBC, RBC, hemoglobin, red cell distribution width, platelet count, office mean BP, and 24-h mean BP; Model 3 adjusted for the parameters in Model 2 plus hs-CRP, plasma renin activity, aldosterone, and angiotensin II. The missing values were recorded, and the rate missing was less than 2% (Supplementary Material). We also used restricted cubic plots to investigate the influence of the BP decline rate on the eGFR decline. All statistical analyses were conducted using SAS 9.3 (SAS, Cary, NC, USA). *P* < 0.05 was considered statistically significant.

## SUPPLEMENTARY MATERIAL

Supplementary Tables
