# Supplementary material for: Nighttime blood pressure decline as a predictor of renal injury in patients with hypertension: a population-based cohort study
Source: Aging (Albany NY). 2019 Jul 5;11(13):4310–22. doi: 10.18632/aging.101873 (PMC6660036; doi:10.18632/aging.101873)
Supplement: Supplementary Tables [file aging-11-101873-s001.docx]

**Table S1. Univariate logistic regression of GFR change.**

| **Parameters** | **Beta** | **SE** | **Waldχ^2^** | **P** | **OR** | **95%CI** | |
| --- | --- | --- | --- | --- | --- | --- | --- |
| age | 0.046 | 0.008 | 31.655 | <0.0001 | 1.05 | 1.03 | 1.06 |
| sex | 0.169 | 0.128 | 1.749 | 0.186 | 1.18 | 0.92 | 1.52 |
| BMI, kg/m2 | -0.064 | 0.019 | 12.073 | 0.001 | 0.94 | 0.90 | 0.97 |
| Waist circumference | 0.002 | 0.007 | 0.093 | 0.761 | 1.00 | 0.99 | 1.02 |
| Smoking | 2.265 | 0.260 | 75.909 | <0.0001 | 9.63 | 5.79 | 16.03 |
| Drinking | 0.017 | 0.086 | 0.038 | 0.845 | 1.02 | 0.86 | 1.20 |
| Physical exercise | 0.013 | 0.019 | 0.487 | 0.485 | 1.01 | 0.98 | 1.05 |
| Triglyceride, mmol/dL | -0.032 | 0.119 | 0.073 | 0.788 | 0.97 | 0.77 | 1.22 |
| LDL-cholesterol, mmol/dL | 0.115 | 0.075 | 2.381 | 0.123 | 1.12 | 0.97 | 1.30 |
| HDL-cholesterol, mmol/dL | -1.016 | 0.290 | 12.319 | 0.000 | 0.36 | 0.21 | 0.64 |
| Total cholesterol, mmol/dL | 0.104 | 0.009 | 6.345 | 0.012 | 1.11 | 1.09 | 1.13 |
| Fasting glucose, mmol/dL | 0.097 | 0.095 | 1.041 | 0.308 | 1.10 | 0.92 | 1.33 |
| Postprandial glucose, mmol/dL | 0.023 | 0.043 | 0.292 | 0.589 | 1.02 | 0.94 | 1.11 |
| Uric acid, mmol/L | 0.028 | 0.005 | 33.296 | <0.0001 | 1.03 | 1.02 | 1.04 |
| Urinary albumin-creatinine ratio, mg/mmol | 0.002 | 0.001 | 3.046 | 0.081 | 1.00 | 1.00 | 1.01 |
| Urinary albumin-creatinine ratio, mg/mmol | 0.024 | 0.003 | 83.979 | <0.0001 | 1.02 | 1.02 | 1.03 |
| Blood urea nitrogen, mmol/L | 0.211 | 0.057 | 13.978 | 0.000 | 1.24 | 1.11 | 1.38 |
| ALT, U/L | -0.005 | 0.003 | 3.249 | 0.072 | 1.00 | 0.99 | 1.00 |
| AST, U/L | -0.002 | 0.006 | 0.118 | 0.731 | 1.00 | 0.99 | 1.01 |
| White blood cell, ×10^9^ | 0.00513 | 0.0446 | 0.0133 | 0.9083 | 1.005 | 0.921 | 1.097 |
| Red blood cell, ×10^12^ | -0.1928 | 0.1696 | 1.2922 | 0.2557 | 0.825 | 0.591 | 1.15 |
| Hemoglobin, g/L | -0.00225 | 0.00495 | 0.2074 | 0.6488 | 0.998 | 0.988 | 1.007 |
| Red cell distribution width, % | -0.0416 | 0.0902 | 0.2126 | 0.6447 | 0.959 | 0.804 | 1.145 |
| Platelet count, ×10^9^ | -0.00307 | 0.00149 | 4.2283 | 0.0398 | 0.997 | 0.994 | 1 |
| High-sensitive CRP, mg/L | 0.0172 | 0.0138 | 1.5479 | 0.2134 | 1.017 | 0.99 | 1.045 |
| Plasma renin activity, ug/lh | 0.00873 | 0.00255 | 11.6796 | 0.0006 | 1.009 | 1.004 | 1.014 |
| Aldosterone, mol/L | 0.2205 | 1.4457 | 0.0233 | 0.8787 | 1.247 | 0.073 | 21.2 |
| Angiotensin II | -0.00888 | 0.00659 | 1.8169 | 0.1777 | 0.991 | 0.978 | 1.004 |
| β-blocker | 0.4445 | 0.0824 | 29.075 | <0.0001 | 1.56 | 1.327 | 1.833 |
| ACE inhibitor | -0.012 | 0.005 | 6.465 | 0.011 | 0.99 | 0.98 | 1.00 |
| A2 blocker | -0.203 | 0.085 | 5.730 | 0.017 | 0.82 | 0.69 | 0.96 |
| Calcium blocker | -0.607 | 0.305 | 3.967 | 0.046 | 0.55 | 0.30 | 0.99 |
| Diuretic | 0.038 | 0.073 | 0.280 | 0.597 | 1.04 | 0.90 | 1.20 |
| Lipid-lowering medication | -0.031 | 0.003 | 83.094 | <0.0001 | 0.97 | 0.96 | 0.98 |
| Baseline eGFR | -1.5916 | 0.0769 | 428.5499 | <0.0001 | 0.204 | 0.175 | 0.237 |
| Office HR | 0.014 | 0.005 | 7.241 | 0.007 | 1.01 | 1.00 | 1.03 |
| Ofice SBP | -0.003 | 0.007 | 0.187 | 0.666 | 1.00 | 0.98 | 1.01 |
| Office DBP | 0.019 | 0.006 | 10.679 | 0.001 | 1.02 | 1.01 | 1.03 |
| Day HR | -0.002 | 0.006 | 0.192 | 0.661 | 1.00 | 0.99 | 1.01 |
| Day mean SBP | -0.015 | 0.007 | 4.482 | 0.034 | 0.99 | 0.97 | 1.00 |
| Day meanDBP | 0.011 | 0.007 | 2.458 | 0.117 | 1.01 | 1.00 | 1.02 |
| Nighttime HR | 0.078 | 0.007 | 133.588 | <0.0001 | 1.08 | 1.07 | 1.10 |
| Nighttime mean SBP | 0.089 | 0.008 | 119.944 | <0.0001 | 1.09 | 1.08 | 1.11 |
| Nighttime mean DBP | 0.034 | 0.007 | 21.862 | <0.0001 | 1.03 | 1.02 | 1.05 |
| night SBP decline rate | -0.348 | 0.021 | 264.998 | <0.0001 | 0.71 | 0.68 | 0.74 |
| night DBP decline rate | -0.012 | 0.007 | 2.699 | 0.100 | 0.99 | 0.98 | 1.00 |
| Nighttime BP decline rate | 0.443 | 0.156 | 8.092 | 0.004 | 1.56 | 1.15 | 2.11 |

**Table S2. Missing baseline parameters in the study.a**

| **Parameters** | N (%) |
| --- | --- |
| Smoking | 4(0.38) |
| Drinking | 8(0.77) |
| Triglyceride, mmol/dL | 9(0.86) |
| LDL-cholesterol, mmol/dL | 10(0.96) |
| HDL-cholesterol, mmol/dL | 10(0.96) |
| Total cholesterol, mmol/dL | 10(0.96) |
| Fasting glucose, mmol/dL | 18(1.73) |
| Postprandial glucose, mmol/dL | 18(1.44) |
| Uric acid, mmol/L | 15(1.92) |
| Urinary albumin-creatinine ratio, mg/mmol | 20(1.92) |
| Blood urea nitrogen, mmol/L | 20(1.92) |
| ALT, U/L | 13(1.25) |
| AST, U/L | 13(1.25) |
| Hemoglobin, g/L | 10(0.96) |
| High-sensitive CRP, mg/L | 16(1.54) |
| Plasma renin activity, ug/lh | 17(1.63) |
| Aldosterone, mol/L | 17(1.63) |
